# Community health workers can improve male involvement in maternal health: evidence from rural Tanzania

**DOI:** 10.3402/gha.v9.30064

**Published:** 2016-01-18

**Authors:** Furaha August, Andrea B. Pembe, Rose Mpembeni, Pia Axemo, Elisabeth Darj

**Affiliations:** 1 Department of Obstetrics and Gynaecology, Muhimbili University of Health and Allied Sciences, Dar es Salaam, Tanzania; 2 Department of Women's and Children's Health, International Maternal and Child Health, Uppsala University, Uppsala, Sweden; 3 Department of Epidemiology and Biostatistics, School of Public Health and Social Sciences, Muhimbili University of Health and Allied Sciences, Dar es Salaam, Tanzania; 4 Department of Public Health and General Practice, Norwegian University of Science and Technology, Trondheim, Norway

**Keywords:** community-based intervention, home-based life saving skills, male involvement, rural Tanzania

## Abstract

**Background:**

Male involvement in maternal health is recommended as one of the interventions to improve maternal and newborn health. There have been challenges in realising this action, partly due to the position of men in society and partly due to health system challenges in accommodating men. The aim of this study was therefore to evaluate the effect of Home Based Life Saving Skills training by community health workers on improving male involvement in maternal health in terms of knowledge of danger signs, joint decision-making, birth preparedness, and escorting wives to antenatal and delivery care in a rural community in Tanzania.

**Design:**

A community-based intervention consisting of educating the community in Home Based Life Saving Skills by community health workers was implemented using one district as the intervention district and another as comparison district. A pre-/post-intervention using quasi-experimental design was used to evaluate the effect of Home Based Life Saving Skills training on male involvement and place of delivery for their partners. The effect of the intervention was determined using difference in differences analysis between the intervention and comparison data at baseline and end line.

**Results:**

The results show there was improvement in male involvement (39.2% vs. 80.9%) with a net intervention effect of 41.1% (confidence interval [CI]: 28.5–53.8; *p* <0.0001). There was improvement in the knowledge of danger signs during pregnancy, childbirth, and postpartum periods. The proportion of men accompanying their wives to antenatal and delivery also improved. Shared decision-making for place of delivery improved markedly (46.8% vs. 86.7%), showing a net effect of 38.5% (CI: 28.0–49.1; *p* <0.0001). Although facility delivery for spouses of the participants improved in the intervention district, this did not show statistical significance when compared to the comparison district with a net intervention effect of 12.2% (95% CI: −2.8–27.1: *p*=0.103).

**Conclusion:**

This community-based intervention employing community health workers to educate the community in the Home Based Life Saving Skills programme is both feasible and effective in improving male involvement in maternal healthcare.

## Introduction

Male involvement in maternal health is recommended as one of the interventions to improve maternal and newborn health (1). As early as 1994, the International Conference on Population and Development (ICPD) in Cairo endorsed the principles of involving men in maternal, neonatal, and reproductive health as well as research that explores the effects of gender roles on health outcomes (2). The action plan stresses the importance of men as partners with shared responsibilities towards sexual and reproductive health. There have been challenges in realising this plan, partly due to gender structures in society and partly due to health system challenges in accommodating men in areas of sexual and reproductive health (3).

Many societies and cultures assume that pregnancy is solely a women's issue and this may have contributed to men not being invited to learn about and engage in matters related to women's and children's health (4). In addition, due to gender structures, men are mostly considered to be the decision-makers in many patriarchal families. They decide when and where the woman can go to access skilled antenatal care (ANC), delivery, and postpartum care, as well as child care (5, 6). This might take the form of deciding how their partners use health services, for example, whether to provide financial support for transportation to access the services (7) or deciding whether a pregnant woman can travel to a referral facility (8–10). At the same time, men, who hold both the economic and decision-making power, are not knowledgeable about complications that can occur during pregnancy and childbirth (11, 12). This can make an impact on women's health when a need arises to take quick actions in seeking expert care (13, 14).

Despite challenges in implementing the ICPD plan of action, there has been some progress made in the increase in male involvement in ANC attendance in areas related to HIV prevention, such as mother-to-child transmission of HIV. This shift in custom has been shown to improve knowledge, adherence to treatment regimen, and rates of exclusive breastfeeding (15–17). Family planning use was also shown to improve in studies where men were included in counselling sessions with their partners (18, 19). Encouraging outcomes have also been recently reported in male involvement in maternal health, including increased access to antenatal and postnatal care (20), reduced maternal smoking, reduced maternal depression, and reduced risk of preterm birth and infant mortality (21–24).

Other interventions, however, have been shown to have negative effects in improving male involvement in reproductive and maternal healthcare. A study completed in Tanzania on counselling for HIV testing, where pregnant women were asked to return to clinic together with their husbands for couple counselling, resulted in women not returning for the second visit (25). Similarly, it was shown in a recent review on male involvement and prevention of mother-to-child transmission (PMTCT) of HIV that uptake of PMTCT was low if unmarried women were obliged to come with their partners (26). Barriers impeding women to reach adequate services have been shown to occur because of gender inequality, such as heavy household workload and having no decision-making power, and inaccessibility to health-related resources, all of which affect child survival (27). Interventions that promoted gender equality and treated men as agents of change, such as making joint decisions and sharing household chores, have been shown to improve interspousal communication, reduce maternal workload, and lead to more involvement in birth preparedness (4, 28, 29). In contrast, if studies are designed in such a way that they consider men's only duty to be a provider of money (i.e. men as instrumental), there is a risk of perpetuating women's disempowerment in relation to health seeking (30, 31). Therefore, it is important to design programmes with strategies that are gender sensitive and can reduce gender inequality (32–35).

In Tanzania, as part of the policy of focused antenatal care (FANC) for the care of pregnant women during ANC, men are encouraged to attend with their spouses. During ANC, the health worker is supposed to discuss birth preparedness and complication readiness (BP/CR) with the couple, as well as general care of the pregnant woman at home (36). In addition, part of the PMTCT national policy is male involvement in voluntary counselling and testing (VCT) and in the overall prevention of perinatal and sexual transmission of HIV. Pregnant women are encouraged to inform their husbands and request that they attend antenatal clinics for HIV-VCT (37). Despite these policies, however, there is no specific strategy to implement this policy.

A way to involve men in sexual and reproductive health is to increase the knowledge of both men and women in the community. Home Based Life Saving Skills (HBLSS) is a programme developed and introduced by the American College of Nurse Midwives (ACNM), which involves the joint training of the pregnant woman, her husband, and other family members (38). The aim is to equip the immediate family members with knowledge in order to recognise life-threatening danger signs, make birth preparedness arrangements, promote health-seeking behaviour, and acquire skills to help the woman if any problem occurs at home. The training encourages dialogue and thus making joint informed decisions when the need for action arises. As such, this is a gender-sensitive programme in that it accommodates both men and women in the intervention.

The aim of this study was to evaluate the effect of HBLSS training in a rural community in Tanzania in improving male involvement in maternal healthcare.

## Materials and methods

### Study design

**Fig. 1:**
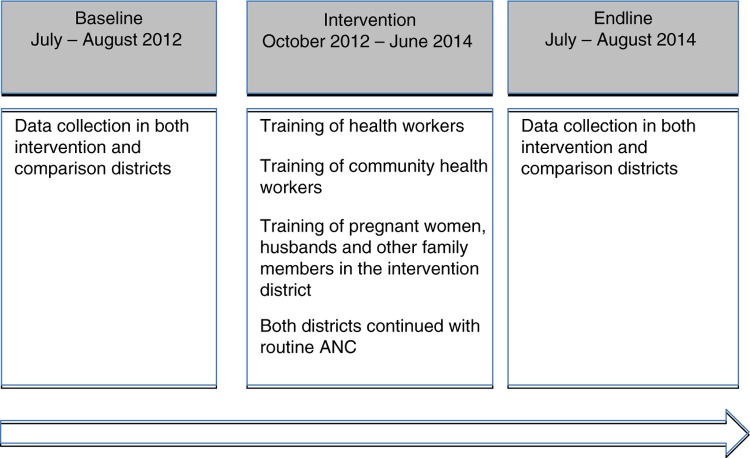
Phases of the intervention.

A quasi-experimental study (non-equivalent group) using pretest–post-test comparison was conducted to evaluate the effect of HBLSS training in the community on male involvement in maternal health in a rural area (Fig. 1).

### Study setting

The study was conducted in two districts in the Pwani Region, Rufiji and Mkuranga, in the south part of Tanzania. Both districts have approximately 222,000 inhabitants, half of them male and half female. Rufiji has a population of 217,274 (108,024 males; 109,250 females), whereas Mkuranga, the comparison district, has a population of 222,921 (104,851 males, 118,070 females). The male literacy rate in the Pwani Region was reported to be 73.2% and the female literacy rate was 66.9% (39). As in Rufiji, agriculture is the main economic activity in Mkuranga District. Common food crops cultivated are cassava, rice, and beans. The main cash crop is cashew nuts. Two hospitals, four health centres, and fifty-two dispensaries serve the population in Rufiji District. Mkuranga has one hospital, five health centres, and fifty-three dispensaries. Clinical officers with 2 years’ training in clinical medicine, registered midwives and enrolled nurses, public health nurses, and maternal and child health aiders provide maternal health services in the health facilities. These include ANC and delivery care services. Men are encouraged to attend the ANC clinic together with their wives (36). Rufiji District was chosen as the intervention district because previous studies completed in the area demonstrated that pregnant women had low awareness of danger signs (40). Mkuranga was chosen as a comparison district due to its comparable population size and socio-demographic characteristics.

### Intervention

The HBLSS education programme covers core topics such as the following: the importance of attending ANC early; recognition of danger signs during pregnancy, delivery, and postpartum and for the newborn; actions to take in case of danger; sharing household chores; making joint decisions on the place to deliver; and identifying skilled care for delivery. Furthermore, the concept of knowledge and actions for BP/CR were included in the educational visits, which relates to saving money, identifying transport, identifying where to go in case of emergency, identifying a skilled attendant, and the preparation of birth kits. Birth kits in this study include buying sterile gloves, surgical blades, a cord tie, and clean clothes. The promotion of health-seeking behaviour to avoid maternal morbidity and deaths was also discussed. The content of the home visits is shown in Table 1. Education was provided to currently pregnant women, their husbands, and other family members. The teaching was done through the use of checklists, skill acquisition, and Take Action cards. These cards consist of images that represent the various problems on one side and which action to take on the other side. The family members are given the cards to keep at the end of each session.

**Table 1:** Training of pregnant women, husbands, and family members on Home Based Life Saving Skills

Visit 1	Visit 2	Visit 3	Visit 4
Importance of attending antenatal care early	Recognition of maternal danger signs and actions to take	Recognition of danger signs and actions to take for early referral	Recognition of maternal and newborn danger signs and actions to take
Recognition of maternal danger signs and actions to take	Birth preparedness and complication readiness	Birth preparedness and complication readiness	Birth preparedness and complication readiness
	Importance of rest and nutrition during pregnancy	Importance of skilled care for delivery and joint decision-making in seeking delivery care	Importance of skilled care for delivery and joint decision-making in seeking delivery care
	Importance of helping the woman with household chores		

Twenty-four health workers from selected health facilities were trained by a master trainer (the first author), who was trained by the ACNM to use the HBLSS concept. The training duration was 1 week. These health workers then trained 66 community health workers (CHWs) for 2 weeks under the supervision of the master trainer. The content of the training included the HBLSS curriculum and BP/CR messages. Refresher training was conducted every 2 months for 1 day to ensure that the education programme was correctly provided. CHWs are trusted men and women in their villages who have completed primary school, are able to read and write, and regularly assist in public health programmes on a voluntary basis. The CHWs were provided with bicycles for easy outreach in this rural community. CHWs identified pregnant women in the community and were asked to make at least four visits to the family throughout the pregnancy. The visits lasted between 60 and 90 minutes. Arrangements were made in advance for a convenient time when the husband and other family members would be available for the educational session. Both the intervention and comparison districts continued to receive the usual services provided by trained health workers at health facilities.

### Sampling and sample size

A two-stage cluster sampling strategy was employed to select a representative sample from the two districts. First, all health facilities (56 in Rufiji District and 58 in Mkuranga District) were listed and then 14 health facilities were randomly selected using the ballot method. In Rufiji District, four health centres and ten dispensaries were randomly selected, whereas in Mkuranga, five health centres and nine dispensaries were randomly selected. Next, two villages forming the catchment population of the health facilities were randomly selected. All men who had partners who had delivered in the last 2 years were selected to participate in the study. The estimated sample size was 1,300 (650 per district). This was based on the assumption of detecting a 10% effect from proportion of male involvement in accompanying spouses of 65.3% (41), considering the power of 80% with 5% significant level, a design effect of 1.5 and non-response rate of 20%. The same procedure was used at baseline and end line, and participants were not necessarily the same during the two surveys.

### Data collection

Before the start of the intervention, a baseline survey was conducted in both districts during 2 months in 2012. An end line survey was conducted 2 years later. Research assistants who had recently graduated from medical school, with experience in conducting interviews, were trained for 5 days before the start of data collection. The questionnaire used in the study was adapted from a questionnaire on BP/CR developed by JHPIEGO, an affiliate of Johns Hopkins University (42). The questionnaire was adapted to fit the Tanzanian context, specifically for the rural areas. A pilot test using 20 questionnaires was conducted in a separate but similar district in the same region. A few alterations were made to the questionnaire following the pilot test. An example of the modifications made is the addition of a question about purchasing birth materials (birth kit), as this was mentioned as a common BP/CR practice. Data were collected under the supervision of the first author. The questionnaire was used to collect information on socio-demographic characteristics, knowledge of danger signs, knowledge and practice of BP/CR components, and place of delivery (Table 2).

**Table 2:** Summary of items used to identify knowledge of danger signs, knowledge on BP/CR, and actions taken

Danger signs				

During pregnancy	During childbirth	During postpartum period	Knowledge of BP/CR	Actions taken on BP/CR
Excessive vaginal bleeding	Excessive vaginal bleeding	Excessive vaginal bleeding	Saving money	Saving money
Convulsions	Convulsions	Convulsions	Identifying transport	Identifying transport
Fever	Delay in placenta delivery	Foul-smelling discharge	Identify where to go in case of emergency	Identifying where to go in case of emergency
Blurred vision	Fever	Difficulty breathing	Identifying skilled birth attendant	Identifying skilled birth attendant
Difficulty breathing	Preterm rupture of membranes	Abdominal pain	Identifying blood donor	Identifying blood donor
Swelling of hands and feet	Prolonged labour	Pain in the perineum	Identifying birth kit	Identifying birth kit
Severe abdominal pain		Body weakness		
Dizziness		Swollen or tender breasts		
Reduced or increased foetal movements				
Ruptured membranes before delivery				

BP/CR, birth preparedness and complication readiness.

### Outcome measures

The main outcome in this study was male involvement. There are no standard criteria in the literature for what is considered to be male involvement. Some authors use criteria such as escorting their partner to ANC or childbirth as a measure of male involvement. Others have used a composite score of several indicators to assess male involvement (43, 44). In our study, we defined male involvement using a composite scoring system. The male involvement score was based on a yes/no answer to the following five components: 1) men escorting their wives to ANC; 2) men escorting their wives to delivery; 3) joint decision-making on where to deliver; 4) knowledge of at least three or more danger signs in each of the phases (pregnancy, childbirth, and the postpartum period), although items to identify knowledge were not ranked according to importance; and 5) at least three BP/CR actions having been taken. A composite index for male involvement was based on the participants having taken three out of the five above actions. The knowledge of danger signs and BP/CR was obtained by asking the participants to mention danger signs without giving any options (Table 2). All variables were measured dichotomously (yes/no). These variables were selected in congruence with previous studies conducted in Uganda and Nigeria that used them to indicate male involvement (13, 43, 44). In addition, the location of the last delivery was recorded. The outcomes were compared before and after the intervention and between the two districts.

### Data analysis

Data were entered into SPSS and cleaned, and in case of any discrepancies the original questionnaire was consulted for cross-checking. Characteristics of the participants were described by using descriptive statistics. An estimated proportion of outcome variables and its variance were calculated according to the cluster sampling design for each group (pretest–post-test intervention) and time point (baseline/end line).

The net intervention effect (NIE) was estimated as the difference between baseline and end line in the intervention group minus the difference between baseline and end line in the comparison group regarding changes in proportions from baseline to end line. This effect is a linear combination of four independent estimates. *p*-values from a *Z*-test and 95% confidence intervals for the intervention effect were calculated based on a normal distribution assumption. A statistically significant effect was considered if *p*<0.05. Statistical analyses were performed with SAS version 9.4 (SAS Institute, Cary, NC, USA).

### Ethical approval

Ethical approval to conduct the study was obtained from the Muhimbili University of Health and Allied Sciences from the Senate Research and Publication Committee (Ref. No. MU/RP/AEC/Vol XIII). Permission to conduct the study in the area was given by the offices of the district executive directors and the village executive officers. The aim of the study was explained to the participants along with their rights in relation to continuing or withdrawing from the study. Informed consent was obtained after participants received the information regarding the aim and objectives of the study. Upon agreeing to participate in the study, participants signed the informed consent form. A thumbprint was accepted for those who could not read or write.

## Results

A total of 1,378 men were interviewed for the study in the intervention district (baseline and end line) and 1,359 in the comparison district (baseline and end line). There was no significant difference in terms of age, marital status, education level, or wife's education level (Table 3).

**Table 3:** Socio-demographic characteristics of participants in the intervention and comparison districts at baseline and end line

	Baseline			End line		
		
	*n*=725	*n*=701		*n*=653	*n*=658	
		
	Intervention, *n* (%)	Comparison, *n* (%)	*p*	Intervention, *n* (%)	Comparison, *n* (%)	*p*
Age of participants			0.832			0.614
<21	28 (3.9)	29 (4.2)		13 (2.0)	5 (0.8)	
21–25	111 (15.3)	88 (12.5)		103 (15.8)	89 (13.5)	
26–30	177 (24.4)	140 (20.0)		110 (16.8)	98 (14.9)	
31–35	143 (19.7)	147 (21.0)		114 (17.5)	150 (22.8)	
>35	266 (36.7)	297 (42.4)		313 (47.9)	316 (48.0)	
Marital status			0.173			0.581
Single	49 (6.8)	35 (5.0)		22 (3.4)	26 (4.0)	
Married/cohabiting	676 (93.2)	666 (95.0)		631 (96.6)	632 (96.0)	
Education level			0.159			0.748
No school	150 (20.7)	146 (21.0)		136 (20.8)	128 (19.5)	
Primary incomplete	64 (8.8)	67 (9.5)		85 (13.0)	47 (7.1)	
Primary completed	450 (62.1)	440 (62.7)		352 (53.9)	415 (63.1)	
Secondary or higher	60 (8.3)	48 (6.8)		80 (12.3)	68 (10.3)	
Wife's education level			0.416			0.693
No school	191 (26.3)	228 (32.5)		194 (29.7)	222 (33.7)	
Primary incomplete	88 (12.1)	65 (9.3)		67 (10.3)	51 (7.8)	
Primary completed	408 (56.3)	379 (54.1)		347 (53.1)	345 (52.4)	
Secondary or higher	38 (5.3)	29 (4.1)		45 (6.9)	40 (6.1)	
Asset quintile			0.207			0.357
A1 (poorest)	21.8	18.5		23.1	18.9	
A2	20.6	22.7		19.5	23.2	
A3	21.7	19.2		20.2	18.7	
A4	18.5	21.3		17.5	21.3	
A5 (least poor)	17.4	18.3		19.7	17.9	

**Fig. 2:**
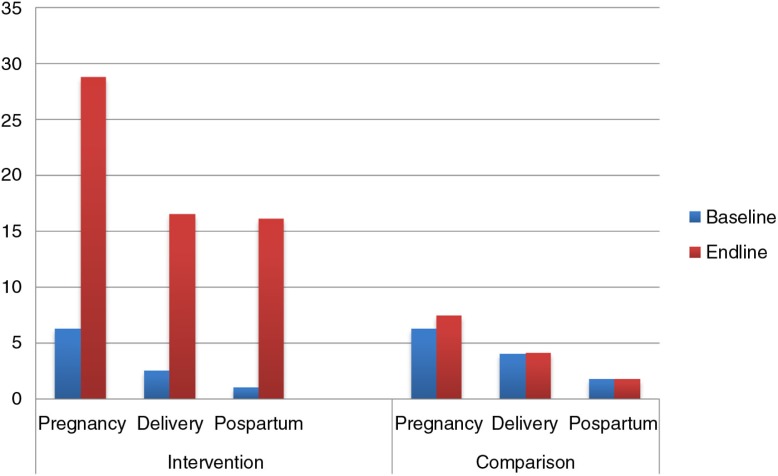
Knowledge of three or more danger signs among men in the intervention and comparison districts at baseline and end line.

Table 4 presents the effect of the intervention on knowledge of danger signs, knowledge of BP/CR and facility delivery. There was significant improvement in knowledge of danger signs across all the phases, during pregnancy, childbirth, and postpartum (Fig. 2). The knowledge of three or more dangers signs during pregnancy increased significantly by a factor of 4 (6.3% vs. 28.8%) with an NIE of 21.3% (95% CI: 13.7–28.9; *p*<0.0001) compared to the comparison district. The proportion of men who mentioned three or more danger signs during childbirth improved in the intervention area (2.5% vs. 16.5%) with an NIE of 13.9% (95% CI: 10.5–17.4; *p*<0.0001). Knowledge of three or more danger signs in the postpartum period also showed improvement in the intervention area compared to the comparison area (1.0% vs. 16.1%) with an NIE of 15.1% (95% CI: 9.2–21: *p*<0.0001). There was improvement in the spouses’ facility delivery in the intervention area as compared to the comparison area, but with non significant estimate of 12.2% (95% CI: −2.8–27.1: *p*=0.103).

**Table 4:** Effect of the intervention on women's facility delivery, knowledge of danger signs, and knowledge of birth preparedness in intervention and comparison districts at baseline and end line

	Intervention (N=798)			Comparison					
					
	*n*=725	*n*=653		*n*=701	*n*=658				
							
	Baseline, *n* (%)	End line, *n* (%)	Difference (%)	Baseline, *n* (%)	End line, *n* (%)	Difference (%)	NIE (%)	95% CI	*p*
Wife's facility delivery	559 (77.1)	579 (88.7)	11.6	492 (70.2)	458 (69.6)	−0.6	12.2	−2.8 to 27.1	0.103
Knowledge of danger signs									
Knowledge of at least 3 during pregnancy	46 (6.3)	188 (28.8)	22.4	44 (6.3)	49 (7.4)	1.1	21.3	13.7 to 28.9	**<0.0001**
Knowledge of at least 3 during childbirth (out of 10)	18 (2.5)	108 (16.5)	14.1	28 (4.0)	27 (4.1)	0.1	13.9	10.5 to 17.4	**<0.0001**
Knowledge of at least 3 during postpartum	7 (1.0)	105 (16.1)	15.1	12 (1.8)	12 (1.8)	0	15.1	9.2 to 21	**<0.0001**
Knowledge of BP/CR									
Knowledge of at least 3	61 (8.4)	223 (34.9)	26.5	54 (7.7)	68 (10.3)	2.6	23.9	19.7 to 26.3	**0.0029**

Significant at *p*<0.01.

Table 5 shows the effect of the intervention on male involvement. The proportion of men who accompanied their spouses to ANC improved (51.9% vs. 72.1%) with an NIE of 16.4% (95% CI: 5.6–27.2; *p*=0.002). For the men accompanying their spouses to delivery, the proportion improved with an NIE of 33.1% (95% CI: 24.1–42.1; *p*<0.0001). Improvement was observed in shared decision-making about place of delivery in the intervention area compared to the comparison area (46.8% vs. 86.7%), showing a net effect of 38.5% (CI: 28.0–49.1; *p*<0.0001). Knowledge of at least three danger signs in each phase during pregnancy, three during childbirth, and three during postpartum increased with a net effect of 27% (CI: 15.3–38.5; *p*<0.0001). The proportion of men who made three or more BP/CR actions increased twofold with an NIE of 26.8% (CI: 15.3–38.2; *p*<0.0001). Overall, the composite index for male involvement (at least three actions taken out of five possible options: escorting the wife to ANC; escorting the wife to delivery; joint discussion on where to deliver; knowledge of at least three danger signs in each phase during pregnancy, delivery, and postpartum; and practice of at least three BP/CR) improved twofold (39.2% vs. 80.9%), showing an intervention effect of 41.1% (95% CI: 28.5–53.8; *p*<0.0001).

**Table 5:** Effect of the intervention on male involvement in intervention and comparison districts at baseline and end line

	Intervention			Comparison					
					
	*n*=725	*n*=653		*n*=701	*n*=658				
							
	Baseline, *n* (%)	End line, *n* (%)	Difference (%)	Baseline, *n* (%)	End line, *n* (%)	Difference (%)	NIE (%)	95% CI	*p*
Escorted wife to ANC	376 (51.9)	471 (72.1)	20.3	321 (53.4)	402 (57.3)	3.9	16.4	5.6–27.2	**0.002**
Escorted wife to delivery	339 (46.8)	526 (80.6)	33.8	289 (48.1)	342 (48.8)	0.7	33.1	24.1–42.1	**<0.0001**
Shared decision-making for delivery	339 (46.8)	566 (86.7)	39.9	251 (41.8)	303 (43.2)	1.4	38.5	28.0–49.1	**<0.0001**
Knowledge of at least 3 danger signs in each of the phases	322 (44.4)	471 (72.1)	27.7	236 (33.7)	226 (34.4)	0.7	27	15.5–38.5	**<0.0001**
Three or more BP/CR actions	158 (21.8)	293 (44.8)	22.9	175 (25.0)	139 (21.1)	−3.8	26.8	15.3–38.2	**<0.0001**
Male involvement score	284 (39.2)	528 (80.9)	41.7	239 (34.1)	228 (34.7)	0.5	41.1	28.5–53.8	**<0.0001**

NIE, net intervention effect; CI, confidence interval; ANC, antenatal care; BP/CR, birth preparedness and complication readiness.

Significant at *p*<0.01.

## Discussion

This study aimed to evaluate the use of HBLSS training by CHWs to improve male involvement in a rural community in Tanzania. Our study shows that there was a significant improvement in male involvement in matters related to maternal health, such as more men accompanying their wives to ANC and for childbirth. Substantial improvement of their knowledge of obstetric danger signs was observed. Couples’ shared decision-making increased as well, as did making preparations for childbirth and for the possibility of obstetric emergency. Although the wives’ facility delivery rate did increase, it was not significant in relation to the comparison district.

Attending ANC is considered to be one of the most important interventions in reducing maternal morbidity and mortality (45). It is the first step that a pregnant woman takes to make contact with the health system during pregnancy. Men have also been encouraged to attend the visits, as they can obtain information regarding the pregnancy. Our intervention improved the proportion of men who accompanied their wives for ANC visits. A study completed in Pakistan showed similar findings (46). The Pakistani study was a community-based randomised trial using volunteers to disseminate audiocassettes and booklets on maternal and newborn danger signs. It demonstrated that more men accompanied their wives to ANC after the intervention. Acquired knowledge on the importance of ANC conveyed by both male and female CHWs using HBLSS could have contributed to this finding. A recent systematic literature review on the impact of male accompaniment to ANC showed that there is an increase in women's knowledge of danger signs, increased facility delivery, and mixed effects on BP/CR. They argued that the effect could be due to the retention of knowledge of essential information and increased communication between couples (47). These findings suggest the importance of male involvement in maternal healthcare and that strategies such as the community-based training of HBLSS should be advocated.

In many societies, men are seen as decision-makers in matters related to health, nutrition, and access to skilled care for their pregnant spouses. With their power to decide, they need to make informed decisions by acquiring knowledge about recognising danger signs, how to prepare for childbirth, and knowing the importance of ANC and institutional delivery care. Furthermore, involving them can contribute to preventing delays in seeking or reaching care (48). It has been observed in other studies that when men possess this kind of information related to pregnancy and knowledge of danger signs, they stress the importance of their wives delivering in a health facility (41, 49). Evidence from our intervention shows that there was significant improvement in the knowledge of danger signs and knowledge on BP/CR. Similar findings were observed in a study conducted in Nepal (29) where pregnant women were counselled in the health facility together with their husbands. In contrast, randomised controlled studies completed in India and South Africa did not show any improvement of men's knowledge of the women's issues if women and their husbands were counselled together (28, 50). The difference in effect between our Tanzanian study and the others from South Africa and India (28, 50) could be related to the fact that only one-third of men who were invited accompanied their spouses to ANC. In addition, the counselling was done in group sessions and not in sessions with individual couples, which could have contributed to the lack of effect. Hence, those who were not exposed to the intervention could have diluted the effect.

The proportion of men who took more BP/CR actions, such as identifying transport, saving money, and identifying a blood donor, improved significantly in our study. A study conducted in Indonesia called *Suami SIAGI* used a multimedia entertainment education intervention. The study, which targeted men, showed similar improvement in men making more preparations for birth (51). A higher proportion of South African women who participated in a randomised controlled trial described receiving more assistance from partners in case of an emergency in terms of arranging transport or taking the woman to the doctor if they had received couples’ counselling at the health facility during ANC (28). In this South African study, although the results showed that men did not significantly improve in knowledge of danger signs, there was improvement in BP/CR. This finding can be explained by the fact that the pregnant women received an enhanced ANC package, were provided with booklets that contained information on ANC, and were encouraged to discuss BP/CR with their partners. In a similar study involving Nepalese men and women in ANC counselling provided at a healthcare facility, no improvement was demonstrated in women making more birth preparations (29). The lack of significant effect in the study in Nepal could be due to the mode of education, which was provided at the health facility. Furthermore, the acquisition of knowledge may not translate into taking desired actions. The mixed effects among the studies indicate the need to perform research that investigates the effect of multiple strategies to improve male involvement.

Our findings show improvement in shared decision-making for determining the place of childbirth. This result is quite encouraging, and it has also been shown elsewhere that male involvement leads to more informed couples, for example, in El Salvador (19). When couples discuss issues together it is easier to make decisions regarding, for example, the use of contraceptives, as was seen in the Malawi Male Motivator project (52) and in India (50). In addition, increased couple communication is associated with increased knowledge of maternal and reproductive health services (53). This is important, as fixed gender norms, where the man is the sole decision-maker, are not reinforced and hence health-seeking behaviour is improved (33).

Facility deliveries increased significantly in the intervention area, but compared to the control district the net effect was not significant. An increase in institutional deliveries has also been observed in other intervention studies where couples are counselled together during ANC visits in hospital settings, for example, in Nepal (29). Contrary to this finding, a community-based intervention in Pakistan (46) did not demonstrate any effect on facility deliveries. A plausible explanation for the non-significant increase in facility deliveries compared to the comparison district in our study could be that the delivery rate at baseline was already high and hence no effect could be detected. In contrast, cross-sectional studies have shown that when men accompany their spouses to ANC there is an increased likelihood of facility delivery (41, 47). More research is needed to rigorously evaluate the mechanisms of including men in ANC and the effect on birth preparedness and facility delivery.

This study has shown that gender norms can be challenged when education is provided in a family setting where both men and women are involved. There was improved joint decision-making, and this aspect is important, especially when seeking care. Joint decision-making may imply good relational and spousal communication. Increased male attendance at ANC may also pose a challenge to the health workers at the health facility. The health facility must be able to accommodate men in terms of infrastructure and privacy. Furthermore, training of health workers on how to counsel couples in terms of understanding that health behaviours and outcomes are a result of social norms and socio-economic relationships could be beneficial. Studies on exploring perceptions of men and women in changing gender norms could help in improving such interventions.

### Strengths and limitations

It is important to use caution while interpreting the results. Because this was a pretest–post-test quasi-experimental study, there was a risk that bias could have been introduced when interpreting the findings. Employing a cluster-randomised trial method could have avoided this bias. The length of time that elapsed between the events and completion of the questionnaire means that participants' responses might be subject to recall bias. Furthermore, the use of a composite score for male involvement that is not universally defined could also be a limitation. It is important to find a common way of describing male involvement in maternal health so as to be able to compare results with other studies.

One of the strengths of this study was the use of both CHWs and health workers. CHWs are also members of the community and therefore are acceptable for providing the HBLSS education. Training of the health workers could have helped them to improve their day-to-day work and hence may provide improved and more appropriate services for ANC.

## Conclusion

Our study has shown that employing CHWs and educating the community using the HBLSS programme improves male involvement in this rural community of Tanzania. The use of CHWs increased men's knowledge of maternal healthcare, improved joint decision-making, and increased accompaniment of spouses to ANC and delivery. It would be possible to scale up this community-based intervention by making home-based ANC visits conducted by CHWs. By increasing health facility deliveries and access to skilled birth attendants, this intervention will make a positive impact on maternal and newborn care.
